# Effects of Technology-Based Perturbation Training During Walking on Physical Function in Stroke, Multiple Sclerosis, and Parkinson's Disease: A Scoping Review

**DOI:** 10.1177/10538135261462948

**Published:** 2026-07-02

**Authors:** Heli Lahtio, Raija Kulmala, Lari Saastamoinen, Sara Suikkanen, Aki Rintala

**Affiliations:** 1 Physical Activity and Functional Capacity Research Group, Faculty of Health Care and Social Services, LAB University of Applied Sciences, Lahti, Finland; 2 Faculty of Health Care and Social Services, LAB University of Applied Sciences, Lahti, Finland; 3 Physical Activity and Functional Capacity Research Group, Faculty of Health Care and Social Services, LAB University of Applied Sciences, Lappeenranta, Finland

**Keywords:** neurorehabilitation, neurological disorder, stroke, multiple sclerosis, parkinson's disease, perturbation

## Abstract

**Background:**

Stroke, multiple sclerosis (MS), and Parkinson's disease (PD) cause balance and walking impairments, which increase the risk of falls and reduce quality of life. Technological advances have enabled novel reactive balance approaches.

**Objective:**

To synthesize the current approaches of technology-based perturbation interventions during walking and their effects on physical function in stroke, MS, and PD.

**Methods:**

A literature search was conducted in PubMed, PEDro, and ScienceDirect for studies published from January 2014 to December 2025, supplemented by citation tracking and grey literature. Eligible studies included interventional studies utilizing technology-based perturbation during walking in stroke, MS, or PD.

**Results:**

Ten studies were included. Participants were diagnosed with stroke (n = 83, 42%), MS (n = 77, 39%), or PD (n = 38, 19%). Most interventions used treadmill-based perturbations to induce slips, trips, or lateral balance instability. Interventions comprised a median of nine sessions over three weeks (25 min per session). Outcomes were grouped into four categories: clinical assessments, patient-reported outcomes, technology-based assessments, and assessments during walking training. Between-group differences compared to similar treatments without perturbation were inconsistent, but within-group improvements in walking speed, balance, and functional mobility were commonly reported. Adverse events were rare, and safety protocols were followed. Considerable heterogeneity in intervention protocols and outcome measures limited synthesis.

**Conclusion:**

Technology-based perturbation training during walking may offer an alternative for improving physical function in stroke, MS, and PD. Evidence of superiority over non-perturbation interventions remains inconclusive due to heterogeneity and small samples. Within-group improvements suggest benefits, warranting standardized protocols and larger high-quality trials.

## Introduction

Stroke, multiple sclerosis (MS), and Parkinson's disease (PD) are neurological conditions that commonly lead to balance impairments and walking disturbances (Cattaneo et al., 2020). Various aspects of balance and mobility, including impairments in overall balance (Camicioli et al., 2023; Cattaneo et al., 2020), walking ability (Cattaneo et al., 2020), reactive balance (Mohamed Suhaimy et al., 2020), postural control (Bohnen et al., 2022; Camicioli et al., 2023), and postural reflexes (Phu et al., 2022), are associated with an increased risk of falls. This elevated risk of falling may subsequently reduce walking activity in daily life. Because walking requires continuous control of dynamic balance to maintain body stability, interventions targeting walking may also enhance balance performance (Lyu et al., 2023). Consequently, both dynamic balance and walking training are essential components of neurological rehabilitation (Lyu et al., 2023; Reichl et al., 2020) and fall prevention (Zhang et al., 2023).

Previous studies on neurological gait rehabilitation have employed a wide range of therapeutic strategies. Traditional approaches typically focus on physical rehabilitation components, including strength training (Boccali et al., 2025; Khalid et al., 2023), balance training, stretching, flexibility training (Boccali et al., 2025), and treadmill training (Boccali et al., 2025; Khalid et al., 2023; Kwok et al., 2022; Lyu et al., 2023; Teodoro et al., 2024). In addition, a variety of complementary methods, such as mental practice (Khalid et al., 2023), task-specific training, the use of ankle foot orthoses (Khalid et al., 2023), functional electrical stimulations (Khalid et al., 2023; Teodoro et al., 2024), virtual reality gait training, robot-assisted training (Khalid et al., 2023; Lyu et al., 2023), obstacle training, action observation training (Kwok et al., 2022; Teodoro et al., 2024), and dual tasking (Teodoro et al., 2024), have also been applied to support gait rehabilitation. Although these conventional strategies have been widely used in neurological rehabilitation for many years (Camicioli et al., 2023; Salari et al., 2021), there has been increasing interest in novel, technology-based therapeutic approaches (Teodoro et al., 2024). One limitation of traditional gait and balance training is that they primarily focus on predictive adaptations (McCrum et al., 2022), with less emphasis on reactive balance in sudden and unexpected situations. However, as falls commonly result from slips and trips during walking, interventions that specifically target the ability to manage these challenges should be considered (Schmidt et al., 2026). Reactive balance interventions have therefore been increasingly investigated among older adults and persons with neurologic conditions (Devasahayam et al., 2023; Kannan et al., 2020; Kim et al., 2022). For example, a network meta-analysis showed that task-specific reactive balance training, including methods such as simulated slips or trips during walking, simulated forward falls, externally applied perturbations, and training on a movable platform, improved reactive balance outcomes in older adults (Kim et al., 2022). Similarly, another meta-analysis reported beneficial effects on fall-related outcomes in both older adults and persons with neurological conditions (Devasahayam et al., 2023). Together, these findings suggest that further development and diversification of training approaches specifically targeting reactive balance are warranted.

One approach for reactive balance training is perturbation training. It consists of repeated external mechanical perturbations which are used to elicit rapid reactions, enabling postural stability to be regained. It aims to improve the ability to recover stability in situations that may lead to falls in everyday life. There are several systems for perturbation training, ranging from floor obstacles and torso pushes and pulls to treadmill-based speed changes, translations, or tilts (McCrum et al., 2022). Perturbation training specifically aims to practice reactive balance by training responses to unexpected external disturbances (McCrum et al., 2022; Schmidt et al., 2026). It practices motor responses to external perturbations by improving intermuscular coordination and response initiation, thereby facilitating rapid balance recovery (Schmidt et al., 2026). Faster postural reflex initiation enables effective balance recovery after a disturbance (Phu et al., 2022).

Perturbation training has been studied among older adults (Gerards et al., 2017; Schmidt et al., 2026), persons with neurological conditions (Brown et al., 2023; Coelho et al., 2022; Kumar et al., 2020; Mohamed Suhaimy et al., 2020; Pigman et al., 2021; van Duijnhoven et al., 2018), and persons with pathological conditions (Phu et al., 2022), but the studies have been highly heterogeneous with variable training protocols. In clinical settings, treadmill-based perturbations or therapist-applied manual perturbations have been utilized (Gerards et al., 2017). Therapist-applied manual perturbations include perturbations with a rolling board which the therapist moves in different directions (Kumar et al., 2020) and therapist-applied manual pushes or pulls (Coelho et al., 2022; McCrum et al., 2022). Perturbation training has been performed in different positions, such as standing (McCrum et al., 2022; Peterson et al., 2025; Phu et al., 2022; Pigman et al., 2021), walking, or during functional transitions such as sit-to-stand (McCrum et al., 2022). It has included methods such as surface movements to different directions, toe-up and toe-down platform tilts, contact with a weighted pendulum, or some combinations, most commonly involving forward and backward translations (Phu et al., 2022). Perturbation training methods have included slip- and trip-like perturbations or perturbations in multiple directions while standing on a treadmill (Schmidt et al., 2026), and slip, trip, or mediolateral shifts while walking (Phu et al., 2022). In addition, computerized movable platforms (van Duijnhoven et al., 2018) have also been used. As illustrated by the variability in intervention types, delivery methods, and equipment described above, perturbation training interventions vary considerably, making it challenging to draw precise conclusions about its effectiveness. However, its importance is supported by the fact that falls typically occur during movement rather than standing (Gerards et al., 2017; McCrum et al., 2022), highlighting the need to incorporate perturbation training into walking. Perturbation training offers an alternative to conventional balance and gait training (McCrum et al., 2022). Furthermore, technology-based training may enhance motivation by providing real-time feedback and gamification elements (Cinnera et al., 2024; Hoffmann et al., 2025).

The aim of this scoping review is to map and synthesize technology-based perturbation interventions during walking. Specifically, this review examines the content of these interventions and their effects on physical function in stroke, MS, and PD. A scoping review design was chosen to identify key concepts and map the emerging evidence on technology-based perturbation training during walking. This review offers practical considerations on how technology-based perturbation can be incorporated into walking exercises.

## Methods

### Study Design

This scoping review maps the content of interventions involving technology-based perturbation training during walking exercises in stroke, MS, and PD. The Joanna Briggs Institute's (JBI) population, concept, and context (PCC) framework (Peters et al., 2020) was applied to formulate the primary review questions, as follows:

1. How is technology-based perturbation training during walking conducted in stroke, MS, or PD?
2. What physical function outcomes have been measured to assess the effects of technology-based perturbation training during walking in stroke, MS, or PD?
3. What effects of technology-based perturbation training during walking have been reported within the outcomes of physical function in stroke, MS, or PD?

### Search Strategy

This review applied the JBI and PRISMA-ScR checklists during the search strategy, screening, and reporting phases (Peters et al., 2020; Tricco et al., 2018). An initial literature search was conducted on 2 January 2025 for studies published from 1 January 2014 to 2 January 2025 on PubMed, PEDro, and ScienceDirect. This was followed by an update search on 5 December 2025 for studies published between 3 January 2025 and 5 December 2025, using the same databases and search terms. To reflect the contemporary state of the field, only studies published from 2014 onwards were considered. This period corresponds with increased availability and clinical implementation of perturbation techniques and a rapid growth in technology-induced rehabilitation research (McCrum et al., 2022). The search strategy used title, abstract, and keyword headings and contained MeSH and general terms for perturbation training and stroke, MS, and PD. The finalized search strings and search filters for each selected database are described in Supplementary File 1. Two researchers (RK & LS) performed the initial searches of the selected databases. Two reviewers (RK & LS) independently screened and assessed the published articles using a predefined strategy that included title, abstract, and full-text screening phases. Any disagreements regarding study eligibility were resolved through discussion between the reviewers, with unresolved cases adjudicated by a third reviewer (HL). The update search was also conducted by two reviewers (AR & HL) following the same principles. In addition, the DANS Data Station Social Sciences and Humanities, DANS Data Station Life Sciences, and ProQuest Dissertations & Theses databases were manually searched for grey literature by one researcher (AR). Grey literature searches were conducted using predefined keywords related to stroke, MS, PD, and perturbation, with identified records screened by one reviewer (AR) using the same eligibility criteria as the database search (Supplementary File 1). Further citation searching was conducted in two phases (Spring and Autumn 2025) by two pairs of researchers (RK & LS; HL & AR), using the reference lists of included studies and applying the original search strategy in Google Scholar.

### Inclusion and Exclusion Criteria

Inclusion criteria were designed according to the PCC (Population, Concept, Context) framework as follows (Peters et al., 2020):

1. Population: Participants diagnosed with stroke, MS, or PD. This review focused on these neurological conditions because of the clinical relevance of perturbation-based training in these populations. Pre-search findings indicated that these conditions commonly present with prolonged impaired gait and gait-related balance, postural instability, and increased risk of falling (Brown et al., 2023; Cattaneo et al., 2020; Mansfield et al., 2015). Limiting the scope to these three conditions also ensured methodological clarity by reducing the heterogeneity of neurological diagnoses.
2. Concept: Technology-based perturbation training during walking exercises to deliver artificial slips, trips, or other disturbance triggers. Perturbation training was defined as any type of technological system capable of delivering perturbations while supporting walking without traditional therapist-applied manual perturbation.
3. Context: Interventional studies (randomized controlled trials [RCTs], pilot-RCT studies, or non-randomized controlled trials [CTs]) in any type of rehabilitation context using technology to trigger perturbations during walking exercises. Given the growing use of technology in perturbation training, we considered it essential to concentrate on only interventional studies to provide more robust data regarding the intervention context, effects, and its implications for clinical practice.

Systematic reviews, meta-analyses, guidelines, diagnostic test accuracy studies, prevalence studies, economic evaluation studies, and qualitative studies were excluded from the review. The selected studies also had to be published in English. If needed, one researcher (HL or AR) contacted the corresponding authors of the study for additional inquiries if the details were not adequately reported to make a final decision based on the criteria.

### Data Extraction

A data charting table was completed by three members of the research group (RK, LS, and AR) who discussed the findings and continuously updated the table in an iterative process. In case of disagreement, a fourth member (HL) was consulted. The data charting table was completed using a predefined list of the studies included after full-text screening. Predefined data included the study details (authors, year, country, study setting, sample size, age, gender, neurological condition, disease duration, and content of the interventions in experimental and control groups), perturbation methods (intensity, duration, technology), other study details (eligibility criteria, dropout rate, adverse events due to perturbation training), and physical function outcomes. If relevant missing data, unreported data, or unclear reporting related to our research questions were observed in the original study, one researcher (AR) contacted the corresponding authors of the included studies for additional information.

### Quality Assessment

To explore the methodological quality of the included studies, we applied the critical appraisal tool by Hawker et al. (2002), which comprises nine domains: 1) Abstract and title, 2) Introduction and aims, 3) Method and data, 4) Sampling, 5) Data analysis, 6) Ethics and bias, 7) Results, 8) Transferability or generalizability, and 9) Implications and usefulness. Each domain was scored on a 4-point scale as good (4), fair (3), poor (2), or very poor (1). Overall study quality was summarized following approaches used in previous studies (Bradley et al., 2018; Hawker et al., 2002; Hudays et al., 2023) by calculating the total domain score and classifying studies as good (> 31 points), fair (26–31 points), poor (11–25 points), or very poor (≤ 10 points). Two research group members (RK & LS) independently assessed the quality of the included studies. In cases of disagreement, a third member (HL) was consulted. Although quality appraisal is not mandatory in scoping reviews, we included it to support the interpretation of clinical findings, as the review focused on the content and effects of intervention training. While not used for study exclusion, the appraisal informed the contextual understanding of the evidence-based literature on the topic.

### Data Synthesis

The extracted data were summarized using descriptive and frequency analyses. Study characteristics included the number of participants (n), personal characteristics comprised age (mean, range) and gender (n, %), and clinical characteristics included neurological condition (n, %) and disease duration (mean, range). Physical function outcomes were grouped into categories and summarized using descriptive analysis within each outcome. All descriptive analyses were conducted using the original datasets to prevent duplicate counting of participants. To explore the effects of technology-based perturbation during walking, a vote-counting analysis was applied to compare findings between experimental and control groups. The vote-counting method assesses the direction of effects by counting studies reporting beneficial (+), non-beneficial (-), or mixed (0) findings without statistical pooling (Hedges & Olkin, 1980).

## Results

### Overview

**Figure 1.**
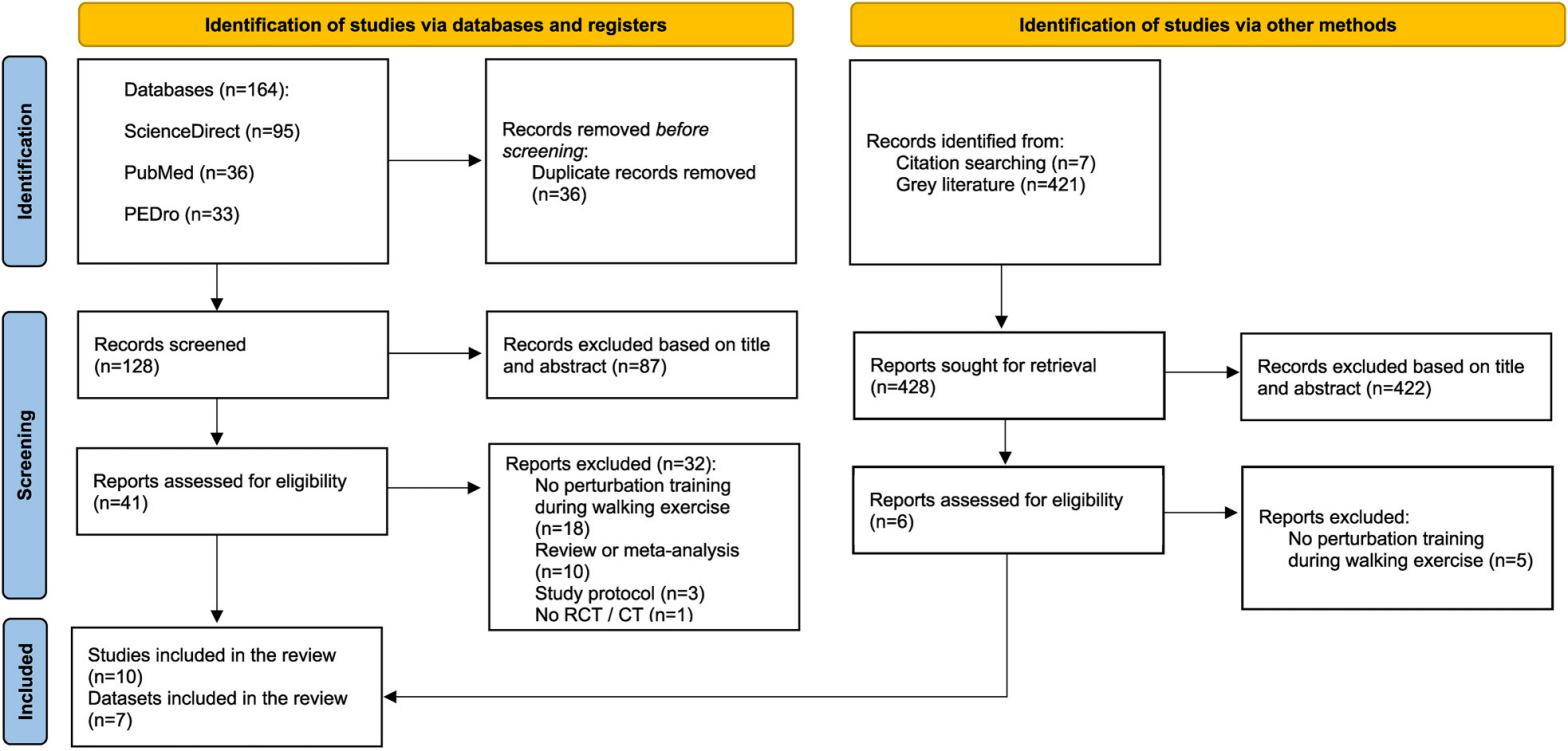
PRISMA flow diagram outlining the study selection process.

After duplicates were removed, a total of 128 studies were identified from searches of electronic databases, citation searching, and grey literature. Based on the title and the abstract, 509 were excluded, with 47 full-text articles to be retrieved and assessed for eligibility. A total of 37 were excluded for the following reasons: 23 did not include perturbation training during walking exercise, 10 were reviews or meta-analyses, three were study protocols, and one was not interventional study. The screening led to a final selection of 10 studies (Esmaeili et al., 2020; Hu et al., 2023; Klamroth et al., 2019; Klamroth et al., 2016; Meyer et al., 2022; Monjezi et al., 2022; Okubo et al., 2023; Steib et al., 2019; Steib et al., 2017; Yang et al., 2019). Three studies (n = 3) were excluded from the descriptive analyses of participant and study characteristics because they used the same dataset (Klamroth et al., 2019; Klamroth et al., 2016; Steib et al., 2019). Of the 10 included studies, four (40.0%) were conducted in Germany, two (20.0%) in the United States, and one (10.0%) each in Canada, China, Iran, and Australia (Figure 1).

### Methodological Quality

The results of the methodological quality assessment are presented in Table 1. Overall, the included studies ranged from good to fair according to the Hawker et al. (2002) appraisal tool. Across individual domains, methodological limitations were primarily observed in sampling and transferability or generalizability, particularly related to small sample sizes and limited reporting of recruitment procedures (e.g., recruitment time, location, and sampling frequency) and study context (e.g., patient characteristics).

**Table 1.** Methodological Quality of the Included Studies.

Author and Year	1. Abstract and Title	2. Introduction and Aims	3. Method and Data	4. Sampling	5. Data Analysis	6. Ethics and Bias	7. Results	8. Transferability Or Generalizability	9. Implications and Usefulness	Total	Quality Score
Esmaeili et al., 2020	4	4	3	2	4	4	4	2	4	31	Fair
Hu et al., 2023	4	4	4	4	4	4	4	3	4	35	Good
Klamroth et al., 2019	4	4	3	2	4	4	4	2	3	30	Fair
Klamroth et al., 2016	3	4	4	2	4	4	4	2	3	30	Fair
Meyer et al., 2022	4	4	3	3	4	3	4	3	4	32	Good
Monjezi et al., 2022	3	3	4	2	4	4	4	2	3	29	Fair
Okubo et al., 2023	4	4	4	3	4	4	4	3	3	33	Good
Steib et al., 2019	4	4	3	3	4	4	4	3	3	32	Good
Steib et al., 2017	4	4	3	4	4	4	4	3	3	33	Good
Yang et al., 2019	4	4	3	2	4	4	3	2	3	29	Fair
Total score of each domain	38	39	34	27	40	39	39	25	33		

### Description of Participants

The eligibility criteria for participation in the experimental group differed slightly between the included studies (Supplementary file 2). Inclusion criteria consisted of a diagnosis of a neurological condition and the ability to stand and walk either on a treadmill, overground, or on a gait mat. Other inclusion criteria were the ability to understand and follow instructions (i.e., no apparent cognitive impairment) and declined balance ability (i.e., a history of at least one fall in the last three months, a Berg Balance Scale cut-off score of ≥ 21 points, or a MiniBESTest score below the lower limit 95% confidence interval of age-specific norms). Exclusion criteria included other skeletal injuries or diseases affecting walking ability or balance, major cognitive deficits, other neurological diseases, active relapses/seizures, or poor ability to tolerate exercises.

The description of participants was analyzed from seven studies (Esmaeili et al., 2020; Hu et al., 2023; Meyer et al., 2022; Monjezi et al., 2022; Okubo et al., 2023; Steib et al., 2017; Yang et al., 2019). The studies involved a total of 198 participants, of whom 80 (41.1%) were female (Table 2). Three (42.9%) studies explored perturbation training during walking in stroke (Esmaeili et al., 2020; Hu et al., 2023; Meyer et al., 2022), three (42.9%) in MS (Monjezi et al., 2022; Okubo et al., 2023; Yang et al., 2019), and one (14.2%) in PD (Steib et al., 2017). The majority of the participants were diagnosed with stroke (n = 83, 42%), followed by MS (n = 77, 39%), and PD (n = 38, 19%). The experimental group included 104 participants, and the control group included 94 participants. Sample sizes were relatively small, ranging from 11 to 18 in the experimental group and 10 to 20 in the control group. Average converted disease duration was 3.5 (SD 3.0) years in stroke (Esmaeili et al., 2020; Hu et al., 2023), 12.0 (SD 2.0) years in MS (Monjezi et al., 2022; Okubo et al., 2023; Yang et al., 2019), and 7.9 (SD 4.0) years in PD (Steib et al., 2017). Regarding disease type and severity, participants with stroke had chronic type (i.e., > 6 months after the stroke event) and were either fully ambulatory or used a walking aid (Esmaeili et al., 2020; Hu et al., 2023; Meyer et al., 2022). The majority of participants with MS had relapsing-remitting MS with low to moderate disability levels according to the Expanded Disability Severity Scale and were able to walk independently with or without a walking aid (Monjezi et al., 2022; Okubo et al., 2023; Yang et al., 2019). Disease severity in PD was reported as mild to moderate according to the Hoehn and Yahr Scale, and participants were able to walk independently without a walking aid (Steib et al., 2017).

**Table 2.** Included Interventional Studies on Technology-Based Perturbation Training During Walking in Stroke, Multiple Sclerosis, or Parkinson's Disease.

Study, Year, Country, and Study Design	N (m/f) EXP/CON	Age mean (SD) EXP/CON	Intervention			Perturbation Technology / Technique	Outcomes and Vote Counting* Results Between EXP/CON (BG) and Within Group (WG) for the Experimental Group Post-Training and Follow-Up
			EXP	CON	Duration		
**Stroke**							
Esmaeili et al., 2020, Canada Pilot-RCT	21 (17/4)	Median (IQR): 58.0 (6.7) / 57.5 (18.0)	Perturbation-based walking training. 9 sessions over 3 weeks (1 session duration not reported). 1 session included warm-up trial with comfortable gait speed, repeated perturbations (2–3 trials for both leg sides) using faster (≥ 140% speed) and slower (between 0–60% speed) belt perturbations, followed by unpredictable perturbations using all types (faster-/slower-belts, paretic/non-paretic side). Faster-belt perturbations simulated trips and slower-belt perturbations simulated slips. Progression: Perturbation intensity increased gradually in each session based on tolerance. Not reported whether the intervention was delivered alongside usual care.	Non-perturbation walking training. 9 sessions of the same number of treadmill sessions at their preferred walking speed, but without perturbations.	3 wk 6 wk FU	Treadmill (Bertec Fit^®^) with integrated perturbation module, safety harness, shoes/barefoot NR	Clinical assessments: *Pre-Post:* 10MWT fast speed (m/s): BG 0 / **WG** **+** **EXP** (mean change = + 0.12, ES 0.60, *p* = .007), WG 0 CON 10MWT self-selected speed (m/s): BG 0 / **WG** **+** **EXP** (mean change +0.15, ES 0.46, *p* = .038), WG 0 CON Mini-BESTest (0–28)**: BG** **+** (*p* = .007) / **WG** **+** **EXP** (mean change = + 3.0, ES = 0.63, *p* = .005), WG 0 CON Non-paretic knee extensors, maximal strength (Dynamometry, Nm): BG 0 / **WG** **+** **EXP** (mean change = + 25.3, ES = 0.58, *p* = .028), WG 0 CON Paretic knee extensors, maximal strength (Dynamometry, Nm): BG 0 / **WG** **+** **EXP** (mean change = + 44.6, ES = 0.59, *p* = .028), WG 0 CON PROMs *Pre-Post:* ABC (0–100): BG 0 / **WG** **+** **EXP** (mean change = + 0.7, ES = 0.52, *p* = .021), WG 0 CON RNLI (0–22): BG 0 / **WG** **+** **EXP** (mean change = -0.5, ES = 0.46, *p* = .040), WG 0 CON *6 wk FU:* ABC (0–100): BG 0 / WG 0 RNLI (0–22): BG 0 / WG 0
Hu et al., 2023, China RCT	33 (17/16)	48.7 (14.8) / 43.1 (16.2)	Perturbation gait and balance training in addition to usual care. 5 × 1.5 h physiotherapy sessions per week for over 4 weeks. Each session included 30 min personalized gait and balance training. Weeks 1–2: 5 min warm-up at comfortable speed, 4 phases of 5 min treadmill speed-dependent gait training, 5 min cool-down at comfortable speed. Weeks 3–4: 5 min warm-up, 4 phases of 5 min perturbation (forward-backwards and lateral transitions) at a comfortable walking speed, 5 min cool-down. Progression: Perturbation intensity was systematically calculated based on forward-backwards and lateral translations (amplitude, velocity, acceleration).	Traditional gait and balance training in addition to usual care. Same number of sessions. Similar training with a 30-min functional task session replacing perturbation sessions. Tasks included standing under various conditions (reduced sensory input, smaller base of support, reaching or catching, responding to unexpected manual perturbations), sit-to-stand, multidirectional stepping, walking, and stair climbing.	4 wk	Treadmill (Balance Tutor) with integrated perturbation module, safety harness or shoes/ barefoot NR	Clinical assessments: 5MWT self-selected speed (m/s): BG 0 / **WG** **+** **EXP** (mean change = + 0.7, *p* = .006), WG 0 CON 5MWT fast speed (m/s): BG 0 / **WG** **+** **EXP** (mean change = + 0.8, *p* = <.001), WG 0 CON 5-STS (s): BG 0 / **WG** **+** **EXP** (mean change = -4.1, *p* = .034), WG + CON (mean change = -3.9, *p* = .038) CST-backward or forward (0/1/2/3): BG 0 / WG 0 TUG fast speed (s): BG 0 / **WG** **+** **EXP** (mean change = -5.0, *p* = .003), WG 0 CON TUG self-selected speed (s): BG 0 / WG 0 PROMs ABC (0–100): BG 0 / WG 0
Meyer et al., 2022, United States Pilot-qRCT	14/15 (24/6)	57.5 (14.2) / 57.8 (13.0)	Walking (anterior /posterior) and stance (left/right) training using a body weight support system with a perturbation module, in addition to usual care. 8 sessions over 2 weeks, 4 sessions per week. Training included various balance exercises using a body weight-supported gait system, such as marching, side-stepping, backward walking, step touches, and lunges. The group also practiced overground walking, stair climbing, and sit-to-stand transitions using the same system. During training, the system delivered balance perturbations in lateral, forward, and backward directions. Progression: Participants started at perturbation level 1 and progressed up to a max. perturbation level of 10 through the course of the study. The amount of force was preset by the manufacturer and the intensity or force was based on the participants’ progress and observational analysis by the therapist. If no proper balance reaction occurred, the level was raised until one appeared.	Group 1: Walking (anterior /posterior) and stance (left/right) training using a body weight support system without a perturbation module, in addition to usual care. 8 sessions over 2 weeks. Exercises were identical but without perturbation. Group 2: Usual care	2 wk	Body weight-supported gait system with integrated perturbation module (ZeroG Gait and Balance System and TRiP integration), safety harness, shoes/ barefoot NR	**Comparison between EXP and similar treatment without perturbation (Group 1):** Clinical assessments: BBS (0–56): BG 0 / **WG** **+** **EXP** (mean change = + 17.9, SD = 8.6, *p* < .001), **WG** **+** **CON** (mean change = + 15.1, SD = 5.6, *p* < .001) PROMS: ABC (0–100): BG 0 / **WG** **+** **EXP** (mean change = + 20.9, *p* = .0006), **WG** **+** **CON** (mean change +20.6, *p* = .0007) mFIM ambulation (13–91)**: BG** **+** (group mean difference post-score = 0.63, *p* = .049) **/ WG** **+** **EXP** (mean change = + 3.9, *p* < .0001), WG + CON (mean change = + 3.5, *p* < .0001) **Comparison between EXP and usual care (Group 2):** Clinical assessments: BBS (0–56): **BG** **+** (group mean difference post-score = + 10.0, *p* < .0001) / **WG** **+** **EXP** (mean change = + 17.9, SD = 8.6, *p* < .001), **WG** **+** **CON** (mean change = + 15.1, SD = 5.6, *p* < .001)
**Multiple Sclerosis**							
Monjezi et al., 2022, Iran RCT	17/17 (28/34)	38.1 (9.5) / 35.1 (8.0)	Perturbation-based balance training in addition to usual care. 3 × 20-min training per week over 4 weeks (in total of 12 training sessions). One session included 12 min of walking and 8 min of standing. After 3 min, 30 backward perturbations were delivered during the remaining 9 min of walking. Walking pace 0.8 km/h and gradually raised the speed in 0.1 km/h intervals until the person stated the current speed to be their preferred speed. Progression: When the participant wished to be more challenged, intensity was increased by 1 Newton to the maximal level, wherein balance could be recovered independently.	Similar exercise without perturbation in addition to usual care.	4 wk	Treadmill (DK City) with a waist-pull perturbation system, safety harness, barefoot	Clinical assessments: 10MWT self-selected speed (m/s): BG 0 / **WG** **+** **EXP** (mean change = + 0.13, ES = 0.81, *p* = .004), WG CON 0 BBS (0–56): BG 0 / **WG** **+** **EXP** (mean change = + 2.0, ES = 0.84, *p* = .004) TUG fast speed (s): BG 0 / **WG** **+** **EXP** (mean change = -1.3, ES = -0.73, *p* = .006), WG CON 0 PROMs: ABC (0–100%): BG 0 / **WG** **+** **EXP** (mean change +14.1, ES = 0.98, *p* = .001), **WG** **+** **CON** (mean change +11.5, ES = 0.61; *p* = .023) Fall rate (number per person, self-report): BG 0 Number of falls (self-report): BG 0 Technology-based assessments (force plates): Latency of postural responses to external perturbations, MCT (ms): **BG** **+** (ES -0.92, *p* = .036) **/ WG** **+** **EXP** (mean change = -15.9, ES = -0.99, *p* = .001), WG CON 0
Okubo et al., 2023, Australia RCT	14/16 (11/19)	55.7 (12.8) / 47.1 (12.8)	Reactive balance training. 2 × 50-min training over 1 wk. Simulations of trips and slips using a specialized 10-meter trip and slip during walking. Each participant experienced 24 trip perturbations and 24 slip perturbations in random order. Progression: Progressive unpredictability, at least 7 washout trials and 4 breaks. Not reported whether the intervention was delivered alongside usual care.	Sham training. Same training where slips were replaced by stepping over foam obstacles during walking.	1 wk	10-meter walkway (GAITRite mat), with hidden tripping board flipping based on hidden foot detection sensor (device NR), camera system, safety harness, shoes	Clinical assessments: CSRT total time, response time, movement time (ms): BG 0 Hand reaction time (ms): BG 0 Knee extension strength (%): BG 0 Postural sway on foam (mm/30 s): BG 0 PROMs: FES-I (16–64): BG 0 Technology-based assessments (motion sensors): *Pre vs. Recovery steps 1–3:* CoM height (cm), MoS (cm), Step length (cm), xCOM (cm): BG 0 Trunk sway range (deg): **BG** **+** (NR; *p* = .043) *Pre vs Recovery step 4:* CoM height (cm): **BG** **+** (NR; *p* = .018) MoS (cm): **BG** **+** (NR, *p* = .032) Step length (cm): **BG** **+** (NR; *p* = .021) xCOM (cm): BG 0 *Pre vs Recovery step 5:* CoM height (cm): **BG** **+** (NR; *p* = .012) MoS (cm), Step length (cm), xCOM (cm): BG 0 *Post-slip trials:* Approach gait speed (cm/s), cadence (NR), CoM height (cm), MoS (cm), step length (cm), trunk sway range (deg), xCoM (cm): BG 0 Other assessments during walking training: Number of falls (during walking training): BG 0 Number of slip falls: BG 0 Number of trip falls: **BG** **+** (fall rate EXP/CON 0.10/0.43, *p* = .044)
Yang et al., 2019, United States CT	13 (5/8)	48.9 (12.2)	Slip perturbation training during walking. 5 min warm up, 5 × 15-s bouts at a comfortable speed before the first slip. A total of 5 slips were delivered. Progression: Intensity was the same for all participants with an acceleration of 8 m/s^2^, peak slip velocity of 1.6 m/s, and slip distance of 0.16 m. Not reported whether the intervention was delivered alongside usual care.	N/A	1 d	Treadmill (Active step^®^ Simbex) with integrated perturbation module, sensors, camera system, safety harness, shoes/ barefoot NR	Technology-based assessments (treadmill sensors): *Comparison between the first slip and the last (5^th^) slip, EXP:* CoM position, recovery foot touchdown (NR): 0 CoM position, slipping foot touchdown (NR)**: +** (mean change = + 0.22, *p* = .004) CoM velocity, recovery or slipping foot touchdown (NR): 0 Foot landing angle (deg)**: +** (mean change = -6.7, *p* = .019) Knee angle at landing (deg): 0 Stability at recovery foot touchdown: 0 Stability at slipping foot touchdown (NR)**: +** (mean change = + 0.10, *p* = .004) Step length (/bh)**: +** (mean change = -0.049, *p* = .013) Recovery step length (/bh): 0 Trunk angle at landing (deg): 0 Other assessments during walking training: Number of falls (during walking training, %): **+** (mean change = -61.5, *p* = .007)
**Parkinson's disease**							
Klamroth et al., 2019, Germany RCT Same dataset as in Klamroth et al., 2016, Steib et al., 2019, and Steib et al., 2017	18/19 (26/11)	67 (8.2) / 63 (7.8)	Perturbation treadmill training during walking, in addition to usual care. 2 × 30 min training per week for 8 weeks. 1 session included 5 min warm-up and cool-down, and 20 min perturbation training that were delivered by tilting the treadmill platform during walking. Progression: participants’ level of self-perceived exertion (Borg 8–20, target 12–15) and difficulty (Likert scale 1 not difficult to 7 extremely difficult, target ≤ 5).	Conventional treadmill training, in addition to usual care. 1 session of similar treadmill training without tilting or perturbations. Measurements were also taken during walking and 10 min afterwards.	8 wk	Treadmill (H/P/Cosmos) with tilting platform (Zebris Medical GmbH), safety harness, shoes	Clinical assessments: Mini-BESTest anticipatory (0–6): BG 0 Mini-BESTest dynamic gait (0–10): BG 0 Mini-BESTest reactive postural control (0–6)**: BG** **+** (EXP: 4.00/CON: 5.50, *p* < .001) Mini-BESTest sensory control (0–6): BG 0 Pull-Test (0–4): BG 0 TUG fast speed (s): BG 0 TUG dual-task fast speed (s): BG 0 Technology-based assessments (treadmill sensors): COP RMS (mm): BG 0
Klamroth et al., 2016, Germany RCT Same dataset as in Klamroth et al., 2019, Steib et al., 2019, and Steib et al., 2017	19/20 (29/10)	65 (10.3) / 64 (8.5)	Perturbation treadmill training during walking. 1 session à 20 min, including 5 min familiarisation, 3 × 5 training sessions in 3-min intervals. Participants walked on a treadmill that continuously generated small, three-dimensional tilting movements. Measurements were taken during walking and again 10 min afterwards. Progression: Not reported.	Conventional treadmill training. 1 session of similar treadmill training without tilting or perturbations. Measurements were also taken during walking and 10 min afterwards.	1 session	Treadmill (H/P/Cosmos) with tilting platform (Zebris Medical GmbH), safety harness, shoes	Clinical assessments: 10MWT self-selected speed (m/s)**: BG** **+** (ES = 0.41, *p* = .014) **/ WG** **+** **EXP** (mean change = + 0.03, SD = 0.13, ES 0.34, *p* = .049), WG 0 CON Technology-based assessments (treadmill sensors): Balance aCoP (mm^2^): BG 0 / WG 0 EXP, **WG** **+** **CON** (mean change = + 239.5, SD = 376.5, ES 0.49, *p* = .009) Balance vCoP (mm/s): BG 0 / WG 0 Cadence: BG 0 / **WG** **+** **EXP** (ES = -0.34, *p* = .048), WG 0 CON Double limb support (NR): BG 0 / **WG** **+** **EXP** (ES = -0.48, *p* = .005), WG 0 CON Stride length variability BG 0 / (NR): **WG** **+** **EXP** (ES = -0.34, *p* = .048), WG 0 CON Stride time (NR): BG 0 / **WG** **+** **EXP** (ES = -0.33, *p* = .055), WG 0 CON
Steib et al., 2019, Germany RCT (secondary analysis) Same dataset as in Klamroth et al., 2019, Klamroth et al., 2016, and Steib et al., 2017	18/20 (27/11)	67 (8.2) / 63 (7.9)	Same intervention as in Klamroth et al., 2019				Technology-based assessments: *8 wk treadmill adaptations (treadmill sensors):* Stance time (% gait cycle): BG 0 / **WG** **+** **EXP** (estimate = -0.06, SE = 0.03, ES = -0.34, *p* = .04), WG 0 CON Stance time asymmetry (%): BG 0 / WG 0 Stance time CV (%): BG 0 / **WG** **+** **EXP** (estimate = -0.04, SE = 0.01, ES = -0.56, *p* < .001), **WG** **+** **CON** (estimate = -0.05, SE = 0.01, ES = -0.70, *p* < .001) Step length asymmetry (%): BG 0 / WG 0 Step time asymmetry (%): BG 0 / WG 0 Stride length (cm): BG 0 / **WG** **+** (estimate = 0.39, SE = 0.17, ES = 0.39, *p* = .02), WG 0 CON Stride length CV (%): BG 0 / **WG** **+** **EXP** (estimate = -0.16, SE = 0.03, ES = -0.75, *p* < .001), **WG** **+** **CON** (estimate = -0.17, SE = 0.03, ES = -0.84, *p* < .001) Stride time (ms): **BG** **+** (estimate = 4.77, SE = 2.38, ES = 0.33, *p* = .05) **/ WG** **+** **EXP** (estimate 4.40, SE = 1.74, ES = 0.41, *p* = .02), WG 0 CON Stride time CV (%): BG 0 / **WG** **+** **EXP** (estimate = -0.15, SE = 0.03, ES = -0.81, *p* < .001), **WG** **+** **CON** (estimate = -0.12, SE = 0.03, ES = -0.71, *p* < .001) Swing time (% gait cycle): BG 0 / **WG** **+** **EXP** (estimate =0.06, SE = 0.03, ES = 0.34, *p* = .04), WG 0 CON Swing time asymmetry (%): BG 0 / WG 0 Swing time CV (%): BG 0 / **WG** **+** **EXP** (estimate = -0.10, SE = 0.03, ES = -0.56, *p* < .001), **WG** **+** **CON** (estimate = -0.11, SE = 0.03, ES = -0.69, *p* < .001) *8 wk overground gait adaptations (motion sensors):* Gait velocity (m/s): BG 0 / WG 0 Stance time (% gait cycle): BG 0 / **WG** **+** **EXP** (estimate -0.43, SE = 4.70, ES = -1.32, *p* = .02), WG 0 CON Stance time asymmetry (%) & stance time CV (%): BG 0 / WG 0 Stride length (cm), stride length asymmetry (%) & Stride length CV (%): BG 0 / WG 0 Stride time (ms), stride time asymmetry (%) & Stride time CV (%): BG 0 / WG 0 Swing time (% gait cycle): BG 0 / **WG** **+** **EXP** (median = 0.43, range = 4.70, ES = 1.32, *p* = .02), **WG** **+** **CON** (median 0.05, range = 3.95, ES = 0.18, *p* = .04) Swing time asymmetry (%): BG 0 / WG 0 Swing time CV (%): **BG** **+** (ES = 0.82, *p* = .02) / WG 0 *3 mo FU treadmill gait adaptations (treadmill sensors):* Stance time (% gait cycle): BG 0 / **WG** **+** **CON** (median = -0.41, range = 2.98, ES = -1.03, *p* = .05) Stance time asymmetry (%): BG 0 / WG 0 Stance time CV (%): **BG -** (ES = 0.85, *p* = .02) / WG 0 EXP, WG - CON (median = 0.22, range = 1.68, ES = 1.63, *p* = .01) Step length asymmetry (%): BG 0 / WG 0 EXP, WG + CON (median = -1.33, range = 15.78, ES = -1.47, *p* = .02) Step time asymmetry (%): BG 0 / WG 0 Stride length (cm), stride length CV (%), stride time (ms), stride time asymmetry (%) & Stride time CV (%): BG 0 / WG 0 Swing time (% gait cycle): BG 0 / **WG** **+** **CON** (median = 0.41, range = 2.98, ES = 1.03, *p* = .05) Swing time asymmetry (%): BG 0 / WG 0 Swing time CV (%): **BG -** (ES = 0.82, *p* = .03) / WG 0 EXP, WG + CON (median = 0.39, range = 3.59, ES = 1.43, *p* = .01)
Steib et al., 2017, Germany Pilot-RCT Same dataset as in Klamroth et al., 2019, Klamroth et al., 2016, and Steib et al., 2019	18/20 (27/11)	67 (8.2) / 63 (7.9)	Same intervention as in Klamroth et al., 2019, and Steib et al., 2019.				Clinical assessments: 2MWT (m): BG 0 / WG 0 6MWT self-selected speed (m/s): BG 0 / **WG** **+** (EXP mean change = + 0.05, SD = 0.12, ES = 0.25; CON mean change = + 0.05, SD = 0.11, ES = 0.24; within-group *p* = .009) 6MWT fast speed (m/s): BG 0 / WG 0 Mini-BESTest all subdomains (0–28): BG 0 / WG 0 TUG fast speed (s): BG 0 / WG 0 PROMs ABC (0–100): BG 0 / WG 0 Technology-based assessments (treadmill sensors): CoP sway, eyes closed or open (velocity mm/s & RMS mm): BG 0 / WG 0 Treadmill training speed (m/s): BG 0 / **WG** **+** (EXP mean change = + 0.2, SD = 0.1, ES = 0.95; CON mean change = + 0.2, SD = 0.1, ES = 0.85; within-group *p* < .001) 3 mo FU: NS all outcomes

N = Number of participants; EXP = Experimental group; CON = Control group; m = Males; f = Females; SD = Standard deviation; BG = Between-group; WG = Within-group improvements for the experimental group; RCT = Randomized Controlled Trial; wk = Week; MCT = Motor Control Test; ms = Milliseconds; ES = Effect Size; *p* = P-value; n = Number; TUG = Timed-Up-and-Go test; 10MWT = 10 Meter Walk Test; BBS = Berg Balance Scale; ABC = Activities-Specific Balance Confidence Questionnaire; MoS = Margin of Stability; cm = Centimeter; NR = Not Reported; CoM = Center of Mass; Deg = Degree; CSRT = Choice Stepping Reaction Time; FES-I = Falls Efficacy Scale-International; /bh = Calculated based on Body Height; m/s = Meters per second; m/s^2^ = Meters per second squared; m = Meter; IQR = Interquartile Range; FU = Follow-up; mini-BESTest = Balance Evaluation Systems Test; RNLI = The Reintegration to Normal Living Index; 5MWT = 5 Meter Walk Test; CST = Compensatory Stepping Test; 5-STS = 5 Times Sit-to-Stand Test; qRCT = Quasi Randomized Controlled Trial; mFIM = Modified Functional Independent Measure; vCOP = Velocity of Center of pressure; mm/s = Millimeters per second; aCOP = Acceleration of center of pressure; mm^2^ = Millimeters squared; COP = Center of pressure; RMS = Root mean square; 2MWT = 2 Minute Walk Test; CT = Non-randomized Controlled Trial; CV = Coefficient of variation; 6MWT = 6 Minute Walk Test

* = Vote-counting: BG + = in favor of experimental group; BG - = in favor of control group; BG 0 = no statistical difference between the groups; WG + EXP = improvements within experimental group, WG + CON = improvements within control group; WG 0 = no improvements within groups

### Technology-Based Perturbation Training During Walking

The content of the interventions of seven studies was analyzed (Esmaeili et al., 2020; Hu et al., 2023; Meyer et al., 2022; Monjezi et al., 2022; Okubo et al., 2023; Steib et al., 2017; Yang et al., 2019). More detailed information on each study is given in Table 2.

#### Technologies

Five (71.4%) studies utilized treadmill technologies (ActiveStep, BalanceTutor, Bertec, DK City, and H/P Cosmos). A perturbation module was integrated into the treadmill system to deliver sudden slip-related triggers (Yang et al., 2019), slip- and trip-related triggers (Esmaeili et al., 2020), or slip, trip, and lateral triggers (Hu et al., 2023). Other techniques included an in-built system generating three-dimensional tilting movements (Klamroth et al., 2016) and a waist-pull perturbation system used to induce sudden slips during treadmill walking (Monjezi et al., 2022). Two (28.6%) other technologies were a gait mat (GAITRite) using slip and trip directions mounted on the walkway using detection sensors (Okubo et al., 2023) and a ceiling-mounted body-weight support walking system (ZeroG Gait and Balance System with TRiP integration) using slip and trip directions and lateral transitions integrated into the system (Meyer et al., 2022). All training approaches used a harness to ensure safety during the walking training, regardless of the technology used.

#### Duration

Weekly training volume, session frequency, and intensity were heterogeneous both between and within disease conditions. Interventions were delivered over a median (IQR) of three weeks (1.5; 4.0). The median (IQR) number of sessions per week was 3.0 (1.5; 3.5), and the median (IQR) number of total sessions was 9 (5.0; 14.0). The median (IQR) duration of one session was 25.0 (16.3; 30.0) minutes.

#### Content of the Experimental Group

Five (71.4.%) studies reported that the intervention was delivered in addition to usual care (Esmaeili et al., 2020; Hu et al., 2023; Meyer et al., 2022; Monjezi et al., 2022; Steib et al., 2017). The training approaches differed in terms of content within each session. Mostly training included a warm-up period, a training period, and a cool-down period. Heterogeneity was observed in terms of walking pace, number of perturbation periods, and number of perturbations within a session. A positive note was that all studies included progression strategies. However, these differed in many ways, with progression based on: 1) the participant's perception of burden (e.g., on the Borg scale) (Monjezi et al., 2022; Steib et al., 2017), 2) a pre-defined cut-off strategy (e.g., acceleration of 8 m/s^2^, peak slip velocity of 1.6 m/s^2^, and slip distance of 0.16 m) (Yang et al., 2019), or 3) a gradual increase over time (e.g., an increase in walking pace or number of perturbations for each session) (Hu et al., 2023; Meyer et al., 2022; Okubo et al., 2023).

#### Content of the Control Group

The training content was heterogeneous across the included studies. In stroke, technology-based perturbation training during walking was compared to treadmill training without the perturbation module (Esmaeili et al., 2020), overground gait and balance training (Hu et al., 2023), or to a ceiling-mounted body-weight support walking system without a perturbation module (Meyer et al., 2022). In MS, control groups were exposed to either treadmill training without the perturbation module (Monjezi et al., 2022) or sham training in which slips were replaced with fixed obstacles during walking (Okubo et al., 2023). One MS study did not include a control group (Yang et al., 2019). In PD, perturbation training was compared to similar treadmill training without a perturbation module (Steib et al., 2017).

#### Adverse Events

Five (71.4%) studies reported that no adverse events occurred during the interventions (Esmaeili et al., 2020; Hu et al., 2023; Monjezi et al., 2022; Steib et al., 2017; Yang et al., 2019). Only one (14.3%) study reported that two (7%) participants dropped out due to bodily pain induced by the reactive balance training (Okubo et al., 2023). One (14.3%) study did not report whether adverse events occurred or not (Meyer et al., 2022).

## Effects of Technology-Based Perturbation Training During Walking on Physical Function

**Figure 2.**
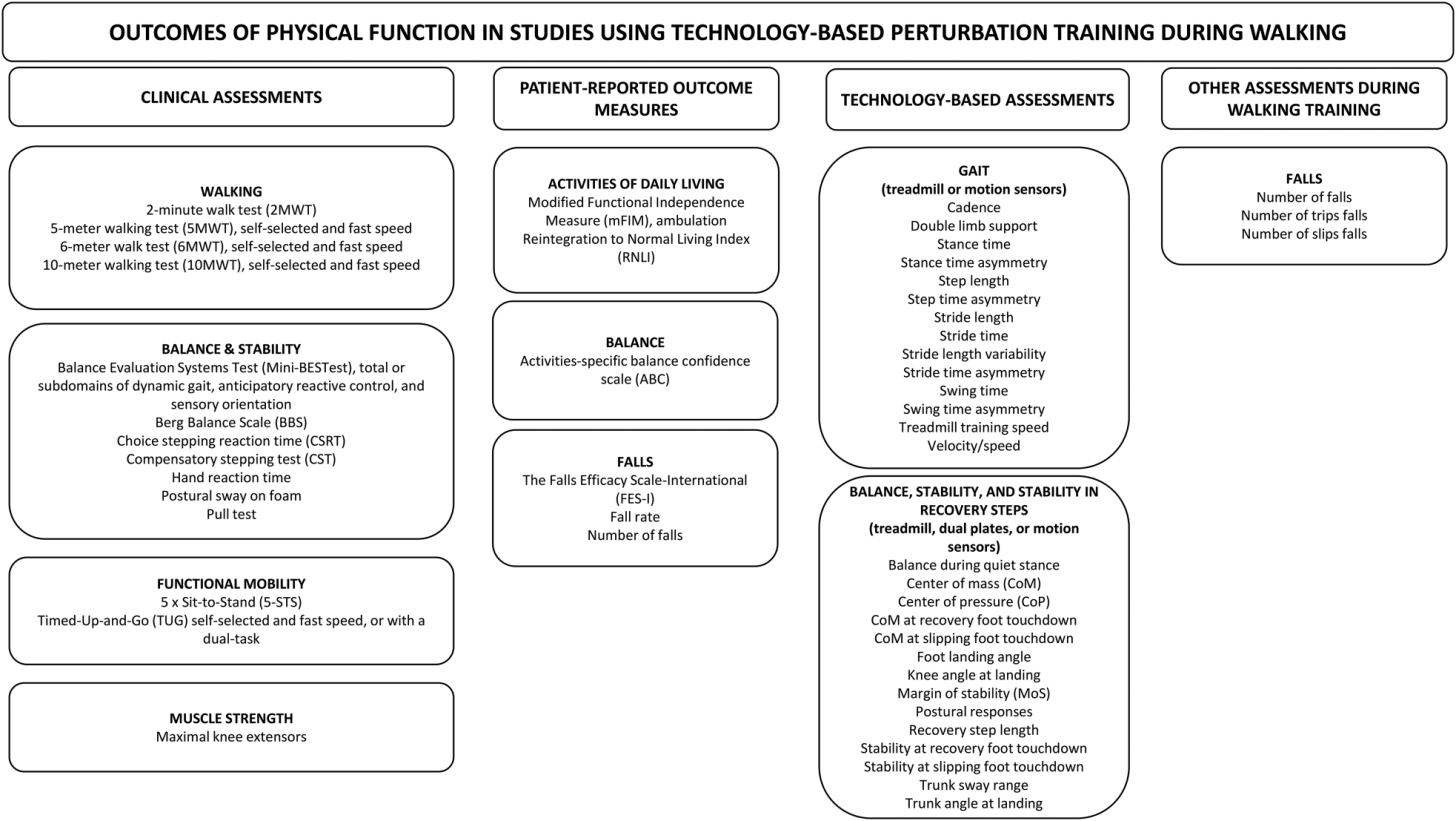
Categorization of physical function outcomes in studies of technology-based perturbation training during walking, including clinical assessments, patient-reported outcomes, technology-based assessments, and other assessments during walking training.

The physical function outcomes were analyzed from all 10 studies (Esmaeili et al., 2020; Hu et al., 2023; Klamroth et al., 2019; Klamroth et al., 2016; Meyer et al., 2022; Monjezi et al., 2022; Okubo et al., 2023; Steib et al., 2019; Steib et al., 2017; Yang et al., 2019). Four categories of physical function assessment were identified (Figure 2):

1. **Clinical assessments** that are relatively easy to administer in clinical practice, including outcomes of walking, balance and stability, functional mobility, and muscle strength,
2. **Patient-reported outcome measures (PROMs)** including self-reported activities of daily living (ADL), balance and falls,
3. **Technology-based assessments** that are measured using a treadmill, gait mat, or other motion sensor analyses, including parameters of gait, balance and stability of recovery steps, and
4. **Other assessments during walking training** that did not fall into the other categories (walking and falls)

No clear between-group differences were identified for any specific outcome across the three neurological disorders. Detailed between-group findings by study population and outcomes are presented in the following paragraphs. More specific perturbation content, as well as within-group findings, are reported in Table 2.

### Stroke

Three (30.0%) out of 10 studies evaluated the effects of perturbation training in stroke (Esmaeili et al., 2020; Hu et al., 2023; Meyer et al., 2022).

#### Clinical Assessments

Balance was assessed in two (66.7%) of three studies, both of which reported statistically significant group differences when perturbation training was compared with similar treatment without perturbations (Esmaeili et al., 2020) or usual care (Meyer et al., 2022). Improvements were observed using the Balance Evaluation Systems Test (Mini-BESTest) after three weeks of training (Esmaeili et al., 2020) and the Berg Balance Scale (BBS) after two weeks (Meyer et al., 2022). Meyer et al. (2022) also compared perturbation training with a control group receiving similar training and did not observe group differences in balance (BBS), in contrast to the findings of Esmaeili et al. (2020) using the Mini-BESTest. No group differences were observed in other outcomes related to balance and stability, walking, functional mobility, or muscle strength.

#### PROMs

One (50.0%) out of two studies reported statistically significant group differences in the activities of daily living (ADL), assessed using the Modified Functional Independent Measure (Meyer et al., 2022). A statistically significant between-group difference was observed after two weeks when perturbation training was compared with similar training without perturbation (Meyer et al., 2022). Similar findings were not observed in another study for ADL measure (the Reintegration to Normal Living Index) after three weeks of training (Esmaeili et al., 2020). No group differences were found in self-reported measures of balance or falls (Esmaeili et al., 2020; Hu et al., 2023; Meyer et al., 2022).

*Technology-based assessments* and *other assessments during walking training* were not examined in studies including participants with stroke.

### MS

Three (30.0%) out of 10 studies evaluated the effects of perturbation training in MS (Monjezi et al., 2022; Okubo et al., 2023; Yang et al., 2019).

#### Clinical Assessments

Two (66.7%) of the three studies assessed clinical outcomes between groups, and no statistically significant differences were reported in any measures related to walking, balance and stability, functional mobility, and muscle strength (Monjezi et al., 2022; Okubo et al., 2023).

#### PROMs

Two (66.7%) of the three studies assessed self-reported outcomes, and no statistically significant differences were found in any balance- or fall-related measures (Monjezi et al., 2022; Okubo et al., 2023).

#### Technology-Based Assessments

Two (66.7%) of the three studies assessed balance and stability, and both reported statistically significant group differences when compared with similar training without perturbations (Monjezi et al., 2022) or sham training (Okubo et al., 2023). Improvements were observed in the postural responses to external perturbations after four weeks (Monjezi et al., 2022) and in the margin of stability after a single session (Okubo et al., 2023). Other gait, balance and stability parameters did not differ statistically between groups.

#### Other Assessments During Walking Training

Outcomes related to falls during perturbation training were assessed in two (66.7%) studies (Okubo et al., 2023; Yang et al., 2019). One study reported statistically significant group differences in favor of the experimental group for the number of trip-related falls after one week of training (Okubo et al., 2023), and the other study did not include a control group for group comparison (Yang et al., 2019). Other statistically significant differences were not reported between groups for the total number of falls or slip-related falls (Okubo et al., 2023).

### PD

Four (40%) out of ten studies evaluated the effects of perturbation training in PD using the same dataset, comparing perturbation training with similar training without perturbations (Klamroth et al., 2019; Klamroth et al., 2016; Steib et al., 2019; Steib et al., 2017).

#### Clinical Assessments

Three (75.0%) out of four studies reported clinical assessments (Klamroth et al., 2019; Klamroth et al., 2016; Steib et al., 2017). Only one (33.3%) study observed statistically significant group differences in walking at self-selected speed using the 10-meter walking test (10MWT) after a single session (Klamroth et al., 2016). In the same participants, Klamroth et al. (2019) reported statistically significant differences in balance using the Mini-BESTest after a single session. No group differences were observed in the same measures of walking (10MWT) and balance (Mini-BESTest), or in other clinical assessments related to walking and functional mobility after eight weeks of training (Steib et al., 2017).

#### PROMs

Only one self-reported balance measure (Activities-specific Balance Confidence scale) was assessed, and no statistically significant group differences were observed after eight weeks (Steib et al., 2017).

#### Technology-Based Assessments

One (25.0%) of three studies reported statistically significant differences in gait parameters using the same dataset comparing perturbation training with similar training without perturbations (Klamroth et al., 2016; Steib et al., 2019; Steib et al., 2017). These differences were observed in stride time during treadmill walking and swing time during overground walking after eight weeks (Steib et al., 2019). Similar outcomes were not statistically different between groups when assessed after a single session (Klamroth et al., 2016). In addition, gait variability appeared to increase only in the control group receiving similar treatment without perturbations when assessed in stance time and swing time (Steib et al., 2019). No other technology-based assessments related to gait or balance showed statistically significant between-group differences.

#### Other Assessments During Walking Training

Only one study measured treadmill training speed as an outcome, and no between-group differences were observed (Steib et al., 2017).

## Discussion

The objective of this scoping review was to map and synthesize current evidence on technology-based perturbation interventions during walking and their effects on physical function in stroke, MS, and PD. This review identified ways to conduct perturbation training using technologies such as a treadmill during walking, and outlined clinical, self-reported, and technology-based assessments that have been used to investigate the effects of such training approaches.

The findings of this review indicate considerable heterogeneity in the use of technology to facilitate perturbation during walking exercises in stroke, MS, and PD. Most of the technology and walking exercises used a treadmill combined with a perturbation technique to simulate slips and trips. Five different treadmill technologies were used, and based on the findings, no single technology outperformed the others. In terms of training, the findings indicate that implementing perturbation training interventions for three weeks, including three sessions of approximately 25 min per week, may be useful in a rehabilitation setting. Similar heterogeneity has also been reported in previous systematic reviews focusing on a broader definition of perturbation techniques in a rehabilitation setting for persons with stroke, PD, and healthy older adults (Brown et al., 2023; Coelho et al., 2022; Hulzinga et al., 2021; Mansfield et al., 2015). Specifically, considerable heterogeneity was observed in training duration, session frequency and length, as well as in the types of devices used (Brown et al., 2023).

The effects of technology-based perturbation training on physical function were assessed using a variety of measures, including clinical outcomes, PROMs, gait and balance analyses (treadmill or motion sensors, or force plates), and assessments during walking training, such as the number of falls due to perturbation-induced slips and trips. No robust effects of technology-based perturbation training for a single outcome were found when compared to similar training without the perturbation feature. Despite this, statistically significant improvements in physical function outcomes were observed within the perturbation group. Our findings align with previous studies that have recommended perturbation interventions as an alternative rehabilitation approach for persons with stroke (Brown et al., 2023) and PD (Hulzinga et al., 2021). This alignment was only partially consistent with previous similar literature reviews on balance in stroke (Alayat et al., 2022), and postural control, walking (Coelho et al., 2022), and decline in falls (Mansfield et al., 2015) among persons with PD. The difficulty of synthesizing such outcomes may be due to the small number of studies investigating technology-based perturbation training while walking, as well as the heterogeneous physical function outcomes. Additionally, previous literature reviews used broader definitions of perturbation, including therapists’ manual perturbation techniques and perturbation triggers while standing rather than walking (Brown et al., 2023; Coelho et al., 2022; Mansfield et al., 2015).

The effectiveness of perturbation training depends on how the training protocol progresses once the person has achieved a desired learning effect or improvement in their postural control, balance, or gait. To our knowledge, no clear synthesis of such progression strategies has been extensively discussed in previous literature reviews (Brown et al., 2023; Coelho et al., 2022; Mansfield et al., 2015). Although progression strategies varied between the included studies, the synthesis of this review identifies three preliminary progression strategies based on the following measures: 1) the person's own perception of burden, 2) the therapist's pre-defined cut-off strategy once the person has achieved a certain number of training sessions, and 3) a gradual increase in walking speed over time after each session. A practical recommendation is that the therapist apply a combination of all three measures. It is important that studies report progression strategies consistently and in detail. If possible, the research community could seek to develop a unified approach, as this could explain why previous reviews are not consistent in terms of the effects of perturbation training.

One positive note among the findings is that safety regulations were aligned across the studies. All included studies reported the use of a safety harness, and no adverse events were reported during the interventions, except in one study that reported bodily pain in two (7%) persons without specification of the affected body region (Okubo et al., 2023). These findings have encouraging practical implications for the application of such technology in rehabilitation settings if safety measures are followed well. Another topic related to safety considerations is which neurological populations perturbation training could be recommended for. Perturbation training could be considered for persons with stroke who have balance deficits (e.g., BSS score ≥ 21 points), are able to walk with or without assistance, and can understand verbal instructions (Esmaeili et al., 2020; Hu et al., 2023; Meyer et al., 2022). Perturbation training has also been successfully implemented in persons with mild to moderate MS (Expanded Disability Status Scale < 6.0), with no disease relapses within the preceding month, and no apparent cognitive impairments (Monjezi et al., 2022; Okubo et al., 2023; Yang et al., 2019). Lastly, perturbation training may be feasible for persons with mild to moderately severe PD (Hoehn & Yahr stage 1–3.5) and at least mild impairment in gait or postural stability (Klamroth et al., 2019; Klamroth et al., 2016; Steib et al., 2019; Steib et al., 2017). The therapist should use extreme caution when considering perturbation training for persons who have skeletal injuries or diseases affecting their walking ability or balance, risk of active relapses or seizures, or symptoms that may limit tolerance for such exercises.

One challenge of perturbation training is the lack of guidelines on how to effectively implement it as part of the rehabilitation in stroke, MS, or PD. Previous reviews provide some evidence on the effectiveness of such training (Brown et al., 2023; Coelho et al., 2022; Hulzinga et al., 2021; Mansfield et al., 2015), but the current evidence is not yet robust enough to define clear, practical guidelines for a targeted perturbation technique. For this reason, this review focused strictly on technology-based perturbation while the person was conducting a walking exercise. Based on the synthesis of this review, several preliminary practical considerations are proposed to guide researchers and therapists when considering the use of treadmill- or gait-based perturbation training during walking as part of a rehabilitation strategy:

- Perturbation training may be incorporated into usual care or delivered as a standalone three-week intervention comprising three sessions per week, each session lasting approximately 25 min.
- Discussions with participants on progression strategies and their documentation are recommended.
- The use of validated balance, postural control, and gait outcomes to measure individual effects is recommended.
- Documenting or reporting falls, either via self-reporting or by the therapist during walking exercises, is recommended.
- Walking training sessions with perturbation should always be divided into warm-up, training, and cool-off periods.
- Familiarization time should be provided for safety reasons before the actual training commences. It is important to document the use of harnesses and footwear.

## Strengths, Limitations, and Recommendations for Future Research

One strength of this review was its focus on interventions solely involving technology-based perturbation training during walking. This review provides a first overview of how technology-based perturbation training is implemented during walking exercises. The justification for such strictness was to provide more nuanced information on perturbation for practitioners and researchers who are more interested in using technologies such as treadmills or other gait mats and perturbation add-on features as an addition to traditional perturbation techniques and walking exercises without technology. We believe that these findings enhance the applicability of such technologies and techniques.

The limitations of this review include the small number of studies as well as extreme heterogeneity in intervention content and small sample sizes within the included studies. This may introduce potential biases, which have also been identified as a challenge in previous literature reviews (Brown et al., 2023; Hulzinga et al., 2021; Mansfield et al., 2015). Another possible limitation is selection bias during the literature screening process, as perturbation training during walking exercises was not always clearly reported in the studies. For example, treadmill exercises with perturbation were also conducted in therapy sessions while standing rather than walking (Peterson et al., 2025; van Duijnhoven et al., 2018). Another potential source of selection bias arises from inconsistencies in the terminology used to describe perturbation training in the current literature (Coelho et al., 2022; Mansfield et al., 2015). Given the nature of scoping reviews, focusing only on interventional studies may be considered a limitation. However, this approach was essential to identify the preliminary content and effects of technology-based perturbation interventions during walking. Finally, clinically meaningful effects could not be determined in the vote-counting analyses, and the preliminary results should be interpreted with caution.

Due to the small number of studies, clinical heterogeneity, and lack of robust between-group effects, further high-quality interventional studies are required to confirm the level of evidence. High-quality RCTs with appropriate power analyses and adequate sample sizes are recommended to establish more robust effects of perturbation interventions compared with other walking exercises, with or without technology. Focusing on disease-specific outcomes, such as fatigue in MS or freezing of gait in PD, could further clarify which types of perturbation training may be beneficial for a particular neurological disorder, taking individual symptom profiles and disease severity into account. Greater consistency in defining and reporting intervention content, safety measures, adverse events, and the progression strategy is needed to improve comparability. In addition, clear reporting of recruitment procedures, in line with CONSORT guidelines, would strengthen transparency and study quality.

## Conclusion

Technology-based perturbation training during walking can be considered an alternative approach in the rehabilitation of persons with stroke, MS, and PD. However, preliminary evidence of superiority over comparable non-perturbation interventions remains inconclusive due to methodological heterogeneity and small sample sizes. Despite this, most studies reported within-group improvements in physical function, indicating that perturbation training may offer benefits for some individuals. Standardized protocols and larger, high-quality interventional studies are needed to clarify clinical effects and support implementation in neurorehabilitation settings.

## Supplemental Material

sj-docx-1-nre-10.1177_10538135261462948 - Supplemental material for Effects of Technology-Based Perturbation Training During Walking on Physical Function in Stroke, Multiple Sclerosis, and Parkinson's Disease: A Scoping Review Supplemental material, sj-docx-1-nre-10.1177_10538135261462948 for Effects of Technology-Based Perturbation Training During Walking on Physical Function in Stroke, Multiple Sclerosis, and Parkinson's Disease: A Scoping Review by Heli Lahtio, Raija Kulmala, Lari Saastamoinen, Sara Suikkanen and Aki Rintala in NeuroRehabilitation

sj-docx-2-nre-10.1177_10538135261462948 - Supplemental material for Effects of Technology-Based Perturbation Training During Walking on Physical Function in Stroke, Multiple Sclerosis, and Parkinson's Disease: A Scoping Review Supplemental material, sj-docx-2-nre-10.1177_10538135261462948 for Effects of Technology-Based Perturbation Training During Walking on Physical Function in Stroke, Multiple Sclerosis, and Parkinson's Disease: A Scoping Review by Heli Lahtio, Raija Kulmala, Lari Saastamoinen, Sara Suikkanen and Aki Rintala in NeuroRehabilitation
