# Supplementary material for: Effects of Technology-Based Perturbation Training During Walking on Physical Function in Stroke, Multiple Sclerosis, and Parkinson's Disease: A Scoping Review
Source: NeuroRehabilitation. 2026 Jul 2;59(1):126–48. doi: 10.1177/10538135261462948 (PMC13477507; doi:10.1177/10538135261462948)
Supplement: Supplementary file 1 [file sj-docx-1-nre-10.1177_10538135261462948.docx]

Supplementary File 1.

Search strategy based on different databases.

**PubMed (n = 36)**

Original search, n = 26

Update search, n = 10

(parkinson* OR stroke OR ”multiple sclerosis” OR ”ms disease”) AND (”perturbation training” OR ”perturbation intervention” OR PBT OR ”reactive balance training” OR ”reactive step training”)

Filters: Clinical trials, RCT, humans, English, published since 2014

**Pedro (n = 33)**

Original search, n = 33

Update search, n = 0

Parkinson* perturb*

Multiple sclerosis perturb*

Stroke perturb*

Filters: Published since 2014

**ScienceDirect (n = 95)**

Original search, n = 71

Update search, n = 24

(parkinson’s disease” OR “parkinson’s disease”) AND (”perturbation training” OR ”perturbation intervention” OR PBT OR ”reactive balance training” OR ”reactive step training”)

(stroke OR ”brain vascular accident” OR “cerebrovascular accident” OR CVA AND (”perturbation training” OR ”perturbation intervention” OR PBT OR ”reactive balance training” OR ”reactive step training”)

(“multiple sclerosis” OR ”ms disease”) AND (”perturbation training” OR ”perturbation intervention” OR PBT OR ”reactive balance training” OR ”reactive step training”)

Filters: Clinical trials, RCT, humans, English, published since 2014

**Grey literature (n = 421)**

*DANS Data Station Social Sciences and Humanities*

Parkinson perturbation (n = 12)

Multiple sclerosis perturbation (n = 138)

Stroke perturbation (n = 20)

Filter: Academic

*DANS Data Station Life Sciences*

Parkinson perturbation (n = 15)

Multiple sclerosis perturbation (n = 68)

Stroke perturbation (n = 89)

Filter: Academic

*ProQuest Dissertations & Theses Global*

Parkinson perturbation (n = 0)

Multiple sclerosis perturbation (n = 76)

Stroke perturbation (n = 3)

Filter: Academic
