# Supplementary material for: Effects of Technology-Based Perturbation Training During Walking on Physical Function in Stroke, Multiple Sclerosis, and Parkinson's Disease: A Scoping Review
Source: NeuroRehabilitation. 2026 Jul 2;59(1):126–48. doi: 10.1177/10538135261462948 (PMC13477507; doi:10.1177/10538135261462948)
Supplement: Supplementary file 2 [file sj-docx-2-nre-10.1177_10538135261462948.docx]

Supplementary File 2.

Eligibility criteria for each included study.

| Study, year, country  Study design | Inclusion criteria | Exclusion criteria |
| --- | --- | --- |
|  |  |  |
| **Stroke** | | |
| Esmaeili et al., 2020, Canada  Pilot-RCT | Chronic  unilateral stroke (> 6 months)  Reduced dynamic balance abilities (MiniBESTest score below the lower limit of the 95% confidence interval of normative data according to age group) with or without a history of falls  Ability to walk on a treadmill without external support  such as handrails or a walking aid for at least 1 min | Hemineglect (more than 6 omissions on the Bells cancellation test)  Major cognitive impairment (Mini-Mental State Examination score below 24/30)  Uncorrected visual deficit  Pathologies other than stroke affecting gait or balance |
| Hu et al., 2023, China  RCT | First-episode stroke  A disease course > 6 months  Perceived balance impairment with impact on daily life (Affirmation to the question: “Do you feel that you experience balance impairment in your daily living?”)  Ability to walk independently with or without an assistive device for at least 10 meters  Gait velocity lower than 0.66 m/s. The gait velocity of 0.66 m/s is a recognized cutoff point for community ambulation capacity | Skeletal injuries or diseases that affected gait and balance ability, including recent fracture of the trunk or lower limbs, severe osteoarthritis or osteoporosis  Vision impairment and neglect  Unable to understand verbal commands (screened using the 3-step command task of the Mini-Mental State Examination)  Unstable blood pressure control  Severe cardiopulmonary disease |
| Meyer et al., 2022, United States  Pilot-qRCT | Stroke diagnosis  ≥ 18 years of age  Score ≥ 21 on the Berg Balance Scale (BBS) during their initial physical therapy evaluation (fair global balance)  Ability to walk with assistance  Able to understand and respond to simple verbal instructions in any language  able to tolerate and actively participate in at least three, 30-minute, weekly sessions | Cognitive deficits that would disrupt the ability to provide informed consent  BBS score <21  Active seizure  Spinal stabilization requiring use of Halo device  Uncontrolled hypertension  Uncontrolled hypotension  Unstable skin structures (i.e., skin grafts and chest tubes)  Unstable rib or lower extremity fractures  Osteoporosis  Active enteric infection control precautions  New limb amputations  Need for >50% high flow oxygen  Bodyweight of more than 450 pounds (204 kg), that is, the structural limitation of the body weight support system |
| **Multiple Sclerosis** | | |
| Monjezi et al., 2022, Iran  RCT | Neurologist-approved diagnosis of relapsing-remitting or secondary-progressive MS  History of at least one fall in the past three months  18–64 years old  Expanded Disability Status Scale (EDSS) <6. | Primary progressive MS  Experienced MS relapses or received exercise interventions for balance in the past three months  Uncorrected visual or auditory deficits  Cognitive impairment confirmed by Mini-Mental State Examination (MMSE) ≤ 24  Using medications that can affect balance and gait  Any neurologic or orthopaedic diagnosis except MS affecting balance and gait |
| Okubo et al., 2023, Australia  RCT | 18 years or older  Living in the community  Confirmed diagnosis of MS  EDSS between 1 and 5.5  No apparent cognitive impairment  Being able to understand and follow test instructions  Stable MS (with or without disease modifying drugs) with no symptom exacerbation in the past 30 days  Exercising for at least 60 min per week (e.g. fitness class, walking, strength training) for the past 3months (the exercise criterion was included as a safety measure, to target the people with MS who were likely to withstand and benefit from exposure to trips and slips) | Unable to walk for five minutes without a bilateral mobility aid  Existing conditions that prevent exercise (e.g. severe pain/spasticity/fatigue, exercise intolerance)  Advised by a medical practitioner not to exercise  History of fracture in the past three years or joint replacement in the past 12 months |
| Yang et al., 2019, United States  CT | A neurologist-confirmed diagnosis of MS  Patient Determined Disease Steps score ≤ 4  Ability to ambulate independently without an assistive device  No significant relapse within the past 8 weeks | Cognitive impairment  Bone mineral loss |
| **Parkinson** | | |
| Klamroth et al., 2016, Germany  RCT | Diagnosis of idiopathic Parkinson’s disease (diagnostic criteria of the German Association for Neurology)  Hoehn & Yahr stage 1–3.5  Unified Parkinson’s Disease Rating Scale (UPDRS) subscore ‘gait’ and/or ‘postural stability’ ≥ 1  Ability to walk independently without an assistive device | Diagnosed with any neurological disease other than Parkinson’s disease  Suffered from any severe cardiovascular or orthopedic condition that would impact performing the assessments and/or intervention  Not able to follow instructions from the assessor and/or care provider due to cognitive impairment |
| Klamroth et al., 2019, Germany  RCT | Same as Klamroth et al., 2016 | |
| Steib et al., 2017, Germany  Pilot-RCT | Same as Klamroth et al., 2016 | |
| Steib et al., 2019, Germany  RCT | Same as Klamroth et al., 2016 | |
